# Rib Cage Deformities Alter Respiratory Muscle Action and Chest Wall Function in Patients with Severe Osteogenesis Imperfecta

**DOI:** 10.1371/journal.pone.0035965

**Published:** 2012-04-27

**Authors:** Antonella LoMauro, Simona Pochintesta, Marianna Romei, Maria Grazia D'Angelo, Antonio Pedotti, Anna Carla Turconi, Andrea Aliverti

**Affiliations:** 1 TBMLab, Dipartimento di Bioingegneria, Politecnico di Milano, Milano, Italy; 2 IRCCS E.Medea, Bosisio Parini (Lc), Italy; Vanderbilt University Medical Center, United States of America

## Abstract

**Background:**

Osteogenesis imperfecta (OI) is an inherited connective tissue disorder characterized by bone fragility, multiple fractures and significant chest wall deformities. Cardiopulmonary insufficiency is the leading cause of death in these patients.

**Methods:**

Seven patients with severe OI type III, 15 with moderate OI type IV and 26 healthy subjects were studied. In addition to standard spirometry, rib cage geometry, breathing pattern and regional chest wall volume changes at rest in seated and supine position were assessed by opto-electronic plethysmography to investigate if structural modifications of the rib cage in OI have consequences on ventilatory pattern. One-way or two-way analysis of variance was performed to compare the results between the three groups and the two postures.

**Results:**

Both OI type III and IV patients showed reduced FVC and FEV_1_ compared to predicted values, on condition that updated reference equations are considered. In both positions, ventilation was lower in OI patients than control because of lower tidal volume (p<0.01). In contrast to OI type IV patients, whose chest wall geometry and function was normal, OI type III patients were characterized by reduced (p<0.01) angle at the sternum (pectus carinatum), paradoxical inspiratory inward motion of the pulmonary rib cage, significant thoraco-abdominal asynchronies and rib cage distortions in supine position (p<0.001).

**Conclusions:**

In conclusion, the restrictive respiratory pattern of Osteogenesis Imperfecta is closely related to the severity of the disease and to the sternal deformities. Pectus carinatum characterizes OI type III patients and alters respiratory muscles coordination, leading to chest wall and rib cage distortions and an inefficient ventilator pattern. OI type IV is characterized by lower alterations in the respiratory function. These findings suggest that functional assessment and treatment of OI should be differentiated in these two forms of the disease.

## Introduction

Osteogenesis imperfecta (OI) is a genetically heterogeneous group of congenital disorders of collagen synthesis characterized by brittle bones leading to different levels of skeletal deformities and frequent multiple fractures.

OI exhibits a broad range of clinical severity, ranging from multiple fracturing in utero to normal adult stature and a low fracture incidence [1], [2]. Although new types of OI syndrome have been recently introduced [3], the traditional classification proposed by Sillence et al. [4], [5] considers four main groups according to the phenotypic variability, i.e. type I, II, III, and IV. OI type I is the mildest form characterized by blue sclerae; perinatal lethal OI type II, also known as congenital OI; OI type III, the most severe non-lethal form characterized by multiple fractures with normal sclera, progressive long bones, spine deformities and short stature; and OI type IV, a moderate form with normal sclerae [5]–[7].

Life expectancy in OI type IV is similar to general population, while in type III is reduced [8] with cardiopulmonary insufficiency and respiratory infections being the leading causes of death in these patients, and their prevention and treatment are important factors for prognosis [9], [10].

Although it is reasonable to postulate that structural alterations of the chest wall, namely spinal and rib cage deformities, may contribute to cardiopulmonary problems in severe OI, few authors reported data on deformities and pulmonary function [11], [12].

The hypothesis of the present paper is that structural modifications of the rib cage due to OI have important consequences on ventilation at rest in terms of chest wall function. To test this hypothesis we noninvasively measure kinematics and volume changes of chest wall in OI patients in two different positions in order to show possible differences between the moderate (type IV) and the most severe (type III) forms and to find possible correlations between chest wall deformities, pulmonary function and ventilatory pattern.

## Methods

### Ethics Statement

The research protocol was approved by the local ethics committee of the Scientific Institute E.Medea, Bosisio Parini, Lc, Italy (approval n. 19/07-CE) and written informed consent was obtained from all patients (or parent). We obtained informed consent from the next of kin on the behalf of the minors/children participants involved in the study.

### Patients

Twenty-two patients affected with osteogenesis imperfecta were recruited for this study. The diagnosis of OI was made on the basis of the following clinical and radiological findings: susceptibility to frequent fractures from the mildest trauma, varying degrees of short stature, progressive skeletal deformities, blue sclerae, dentinogenesis imperfect, joint laxity, early loss of hearing, genetic and/or biochemical analysis with heterozygosity of dominant mutations in one of the two genes (COL1A1 and COL1A2) encoding the chains of type I collagen, with quantitative (mild or moderate forms) or qualitative (severe or lethal forms) defect of the synthesis of type I collagen [2].

Seven patients were classified as type III, the most severe form of OI and 15 patients as type IV.

Twenty six healthy subjects were recruited as control group.

Two type III patients were under nocturnal non-invasive ventilation; 7 patients (5 type III, 2 type IV) were not ambulant; one type IV patient showed basilar impression; one type IV patient had costal fractures secondary of a car crash occurred three months before the study.

### Pulmonary function tests

Measurements of forced vital capacity (FVC), forced expiratory volume in one second (FEV_1_), subdivision of lung volumes (Functional Residual Capacity, FRC; Residual Volume, RV and Total Lung Capacity, TLC) by the nitrogen washout technique were performed (Vmax series 22, SensorMedics, Yorba Linda, CA). Spirometric and lung volumes predicted values were computed using gender, age and height, according to the most commonly used equations of Quanjer et al [13] but also to more recent equations of Kuster et al [14], valid on a wider range of ages.

Nocturnal oxygen saturation (SpO_2_) was measured using a digital pulse oximeter (Nonin, 8500 digital pulse oximeter Quitman, TX). Pulse oxymeter was positioned by specialized personnel and only recordings longer than 8 h were considered as acceptable, otherwise measurements were repeated during the following night.

### Kinematic Analysis

Total and compartmental chest wall volumes were measured by Opto-Electronic Plethysmography (OEP System; BTS, Milan, Italy) [15], [16]. The system, based on eight special infrared video cameras working at a sampling rate of 60 Hz, computes the 3D coordinates of retro-reflective markers placed on the trunk of the subject according to specific anatomical points from clavicles to pubis. When patients were lying supine on the bed, 52 markers were placed over the anterior chest wall surface [17], [18], while an 89 markers configuration was used for measurements in the seated position [16]. Patients and controls were analyzed during three minutes of spontaneous quite breathing in awake diurnal state firstly in seated and successively in supine position.

### Chest wall volumes and ventilatory pattern

Chest wall was divided into three compartments: pulmonary rib cage (RCp), under the action of rib cage muscles, abdominal rib cage (RCa), under the insertional action of diaphragm and abdomen (AB), under the action of the diaphragm and the expiratory abdominal muscles.

**Figure 1:**
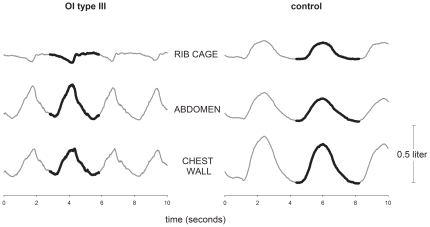
Thoraco-abdominal volume variations during spontaneous breathing. Time courses of the volumes of the rib cage (sum of the pulmonary and abdominal rib cage), abdomen and total chest wall during ten seconds of consecutive breaths at rest in supine position in a representative OI type III patient (left panes) and a representative healthy control subject (right panels). The thick lines highlight a single breath.

The volume of the entire chest wall (V_CW_) and its compartments (V_RCp_, V_RCa_, V_AB_) were noninvasively measured by Opto-Electronic Plethysmography. Figure 1 shows an example of thoraco-abdominal volume traces during ten seconds of spontaneous supine quite breathing of a type III patient and an healthy subject. In this representative example, for clarity, the volumes of the two rib cage compartments are summed to provide total rib cage volume.

From variations of V_CW_ during breathing the ventilatory pattern, in terms of minute ventilation, breathing frequency, tidal volume and percentage contribution of each compartment to tidal volume was determined for each position.

### Chest wall dimensions and deformity

**Figure 2:**
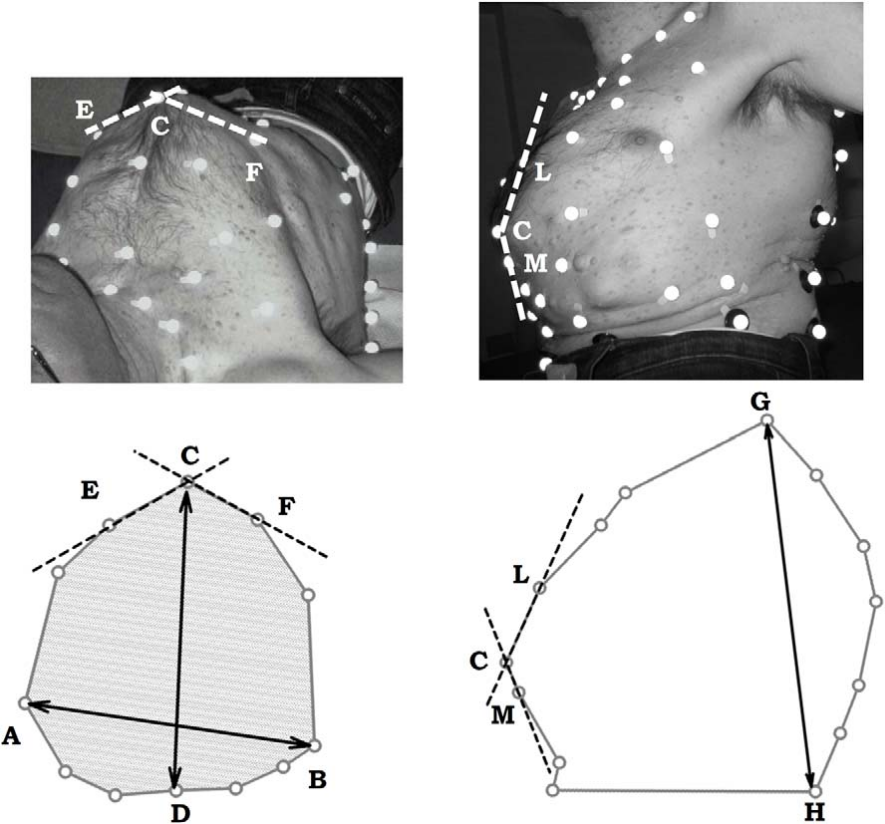
Assessment of chest wall geometry and rib cage deformity by markers' projections. Experimental set-up for the analysis of chest wall volumes via optoelectronic plethysmography in supine (a) and seated (b) position on a representative OI type III patient. c: schematic view of the markers on the transversal plane at the xiphosternal level, in which medio-lateral diameter (distance between markers A and B), antero-posterior diameter (distance between markers C and D), area (grey area) and transversal angle (angle formed between lines CE and CF) are shown; d: schematic view of the markers on the sagittal plane in which trunk height (distance between markers G and H), and sagittal sternal angle (enclosed within lines CL and CM) are shown.

To characterize chest wall geometry several geometrical parameters were calculated from 3D marker coordinates measured by Opto-Electronic Plethysmography in seated position at end-expiration [18]. These included trunk height, chest wall surface and total and compartmental trunk volumes. The latter were obtained as the volume enclosed by the surface obtained by triangulating all the markers. Several additional parameters including medio-lateral and antero-posterior diameter, perimeter and cross sectional area were calculated at five vertical levels (Louis' angle, xiphoid process, lower costal margin, umbilical and iliac crest) (figure 2). In addition, to quantitatively describe the geometrical deformity of the rib cage (‘pectus carinatum’) the angles subtended at the sternal level on the transversal plane (figures 2a and 2c) and on the sagittal plane (figures 2b and 2d) were calculated.

### Thoraco-abdominal asynchronies

Dynamic thoraco-abdominal asynchronies were quantified by determining the phase shift angle (Φ_TA_) between volume variations of V_RC,P_ and V_AB_ waveforms. Φ_TA_ was derived from the ratio of the distance m delimited by the intercepts of V_RC,P_ (y-axis)-V_AB_ (x-axis) loop on a line parallel to the x-axis at 50% of ΔV_RC,P_ divided by ΔV_AB_ (s) and computed as Φ_TA_ = sin^−1^(m/s) [19], [20]. In a similar way, dynamic asynchrony between the two rib cage compartments was calculated as the phase shift angle between V_RC,P_ and V_RC,A_ waveforms (Φ_RC_). By convention a positive angle means that RCp expansion is leading on RCa (or AB) expansion; on the contrary negative angles describe the reverse situation.

Labored Breathing Index (LBI) [21] was calculated as the ratio between the sum of maximal V_RC,P_,V_RC,A_, V_AB_ variations and tidal volume (maximal V_CW_ variation).

### Spinal deformity assessment

Radiographic measurements included standard standing antero-posterior and lateral views of the entire spine. Thoracic scoliosis was measured using the Cobb method. If two curves were present, the largest thoracic curve was used.

### Statistical analysis

Anthropometric, spirometric and lung function data of the three groups were compared using a one-way Analysis of Variance with disease as independent factor. To compare ventilatory pattern data between the groups in the two postures, a two-way Analysis of Variance was performed with posture and disease as independent factors. Post-hoc tests were based on Holm-Sidak method. Data in tables are expressed as mean±standard deviation, in figures as mean±standard error. Significance was determined by p<0.05.

## Results

### Anthropometry, chest wall dimensions and deformity

**Figure 3:**
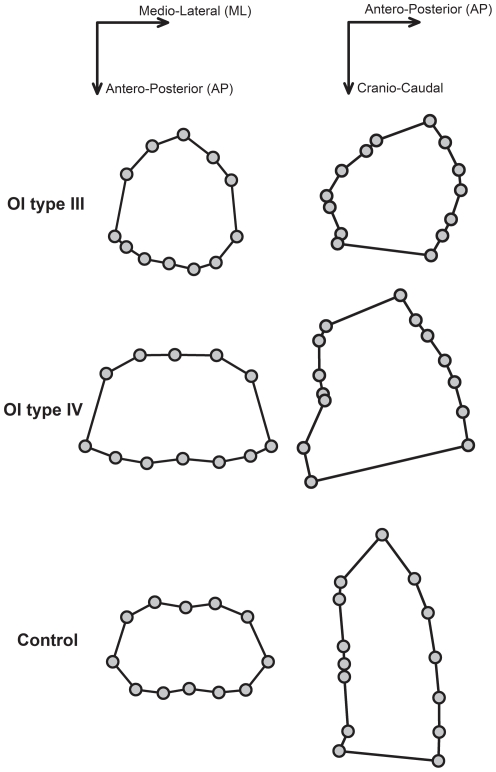
Transversal and sagittal sections of OI type III, OI type IV and controls. Markers' projection on the transversal (left) and sagittal (right) views of the markers (at xiphoid and sternal level, respectively) on three representative subjects: OI type III (top), OI type IV (middle) and healthy control (bottom).

Anthropometric characteristics of OI type III, OI type IV and controls are reported in table 1. Height was different among the three groups, being the lowest in OI type III patients. Weight and body surface area were lower in OI patients than controls. Among all the geometrical parameters, only two resulted different: trunk height (being lower in OI type III patients) and the angle at sternum level, both in sagittal and transversal planes. Patients with OI type III were characterized by lower angles (i.e., pectus carinatum) in contrast with the flatter sternum of both OI type IV and controls (figure 3).

**Table 1:** Osteogenesis imperfecta (OI) patients' and healthy controls characteristics.

	OI type III				OI type IV				Control group		
**Anthropometry**											
Patients n (M/F)		7	(5/2)			15	(8/7)			26	(17/9)
Age (yrs)	26.1	±	16.3		15.9	±	11.4		22.4	±	18.3
Height (cm)	106.6	±	14.7	°°°,*	127.7	±	18.3	°°°	157.0	±	23.2
Weight (Kg)	30.6	±	15.3	°°	39.6	±	17.5	°°	54.2	±	24.4
Body Mass Index (Kg/m^2^)§	27.8	±	13.9		23.2	±	6.3		23.2	±	6.3
Body Surface Area (m^2^)§§	0.89	±	7.00	°°°	1.16	±	15.00	°°	1.52	±	0.45
Trunk volume (L)	11.5	±	5.9		13.7	±	7.7		15.8	±	8.9
Rib cage volume (% trunk volume)	63.7	±	15.1		68.9	±	4.9		71.6	±	3.6
Abdominal volume (% trunk volume)	36.3	±	15.1		31.1	±	4.9		28.4	±	3.6
Trunk Surface (m^2^)	0.28	±	0.11		0.32	±	0.12		0.39	±	0.13
Trunk Height (cm)	18.4	±	5.6	°°°,***	27.6	±	3.8	°°°	40.4	±	6.5
Medio-Lateral Diameter^1^ (cm)	27.7	±	7.9		30.1	±	7.0		31.4	±	5.7
Antero-Posterior Diameter^1^ (cm)	19.3	±	7.0		20.8	±	4.3		19.6	±	4.0
Perimeter^1^ (cm)	0.80	±	0.22		0.85	±	0.19		0.85	±	0.16
Cross sectional area^1^ (cm^2^)	422.5	±	252.4		523.3	±	219.0		515.4	±	199.8
Transversal angle^1^ (degree)	161.6	±	17.5	°°,**	178.3	±	10.5		181.7	±	12.9
Sagittal angle^1^ (degree)	155.8	±	26.6	°°°,***	170.2	±	13.9		170.1	±	12.7
Cobb Angle (deg)^2^	54.7	±	14.9		41.1	±	14.2			n.a.	

Data are expressed as mean±standard deviation.

°°, °°° : p<0.01, p<0.001 (vs control).

*, **,*** : p<0.05, p<0.01, p<0.001 (vs OI type IV).

§ Body Mass Index = *weight* (Kg)/(*height* (m)) ^2^.

§§ Body Surface Area (m^2^) = 0.024265×*weight* (Kg) ^0.5378^×*height* (cm) ^0.3964^.

1 at sternal level.

2 maximal.

n.a. = not available.

All patients were characterized by severe or moderate scoliosis (Cobb Angle>20°). Average Cobb angle values were >40° in both OI types and no differences were observed.

### Pulmonary function

Spirometric parameters, lung volumes and nocturnal saturation data are shown in table 2.

**Table 2:** Osteogenesis imperfecta (OI) patients' pulmonary function test.

	OI type III			OI type IV		
**Spirometry (n)**		5			9	
FVC (L)	1.21	±	0.7	2.31	±	0.7
FVC (% pred)^1^	115.7	±	72.3	96.1	±	26.7
FVC (% pred)^2^	69.6	±	31.5°	86.3	±	24.4
FEV_1_ (L)	0.98	±	0.5	2.04	±	0.6
FEV_1_ (% pred)^1^	91.4	±	50.8	98.1	±	29.1
FEV_1_ (% pred)^2^	59.4	±	26.6°°	84.3	±	24.2
FEV_1_/FVC	81.4	±	5.4	88.4	±	3.8
**Lung Volume (n)**		5			9	
TLC (L)	2.2	±	1.1	3.36	±	1.2
TLC (% pred)^1^	109.33	±	7.5	95.0	±	26.2
TLC (% pred)^2^	120.7	±	27.3	87.3	±	23.8
RV (L)	0.72	±	0.4	0.99	±	0.6
RV (% pred)^1^	86.8	±	14.2	82.0	±	28.8
RV (% pred)^2^	85.1	±	10.8	79.4	±	28.8
RV/TLC (%)	33.0	±	9.7	28.4	±	7.2
FRC N_2_ (L)	0.85	±	0.5	1.44	±	0.6
FRC N_2_ (% pred)^1^	60.6	±	37.9	74.8	±	28.6
FRC N_2_ (% pred)^2^	46.7	±	21.9	59.3	±	17.8
**Nocturnal SpO_2_ (n)**		7			15	
% time with SpO_2_<95%	17.7	±	19.7	7.9	±	13.5
Average SpO_2_ during all night	91.9	±	12.2	96.8	±	2.4
Nadir	85.4	±	8.1	87.7	±	7.3

Data are expressed as mean±standard deviation. FVC: forced vital capacity;

1 Reference values from Quanjer et al, 1993;

2 Reference values from Kuster et al, 2008; FEV_1_: forced expiratory volume in 1 s; TLC: total lung capacity; RV: residual volume; FRC N_2_: functional residual capacity measured by N_2_ washout; SpO_2_: arterial oxygen saturation measured by pulse oxymetry; %pred: percentage of predicted value.

°,°° p<0.05, p<0.01 vs Quanjer et al.

Spirometry and lung volume data were not available on patients younger than 9 years (2 type III and 6 type IV) due to their limited collaboration.

According to the most commonly used equations of Quanjer et al [13], patients showed FVC and FEV_1_ values (expressed as percentage of predicted values, %pred) within the normal range, with data of OI type III unexpectedly higher than predicted. Using more recent equations of Kuster et al [14], FVC (%pred) and FEV_1_ (%pred) became significantly lower in OI patients, particularly in OI type III. No differences were found using the two different predicted equations for TLC, RV and FRC. Finally, although OI type III patients showed a more pronounced tendency to decrease oxygen saturation (SpO_2_) during the night compared to OI type IV, this was not significant.

### Ventilatory pattern

Minute ventilation and its two components, breathing frequency and tidal volume (V_T_), are summarized in table 3. In supine position there were no differences in minute ventilation, while V_T_ and breathing frequency components were different, being lower the former and higher the latter in OI patients. No differences were found between type III and type IV. In the seated position minute ventilation was lower in OI patients than controls due to lower V_T_. Only in OI type III patients, breathing frequency was higher than controls.

**Table 3:** Ventilatory pattern of osteogenesis imperfecta (OI) patients' and controls in seated and supine positions.

	OI type III					OI type IV			Control group		
***Supine position***											
Minute ventilation (L min^−1^)	4.65	±	1.70		5.31	±	1.81		6.41	±	2.83 •••
Breathing frequency (min^−1^)	25.6	±	15.6	°°	22.2	±	5.7	°	17.5	±	4.1
Tidal volume (L)	0.24	±	0.14	°	0.25	±	0.11	°	0.38	±	0.18 •••
***Seated position***											
Minute ventilation (L min^−1^)	4.51	±	1.68	°°°	5.40	±	2.05	°°°	8.42	±	3.29
Breathing frequency (min^−1^)	25.5	±	15.1	°	22.4	±	6.5		18.6	±	5.0
Tidal volume (L)	0.23	±	0.14	°°°	0.25	±	0.09	°°°	0.48	±	0.21

Data are expressed as mean±standard deviation.

°, °°, °°° : p<0.05, p<0.01, p<0.001 (vs control).

••• : p<0.001 (vs seated).

### Chest wall compartment volumes

**Figure 4:**
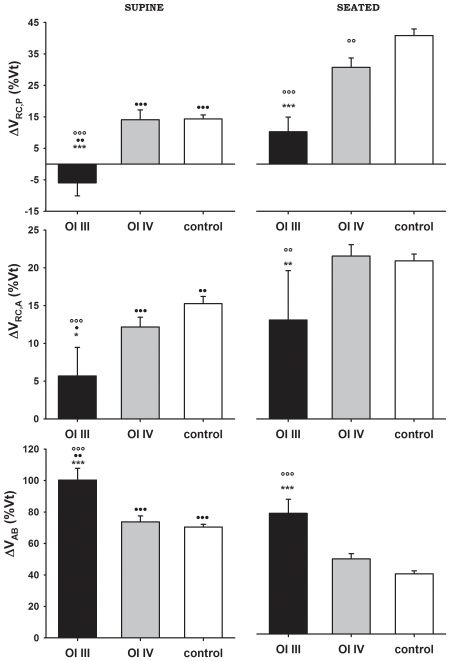
Thoraco-abdominal contribution to tidal volume. Average values ± SE of pulmonary rib cage (top panels), abdominal rib cage (middle panels) and abdominal (bottom panels) percentage contribution to tidal volume in OI type III patients (black bars), OI type IV (grey bars) and control group (white bars) in supine (left panels) and seated (right panels) position. °°, °°° : p<0.01, p<0.001 (vs control); *, **, ***: p<0.05, p<0.01, p<0.001 (vs OI type IV); •,••,•••: p<0.01, p<0.001 (vs seated).

Patients with OI type III were characterized by reduced contributions of rib cage compartments to V_T_ as shown in figure 4. The percentage contribution of V_RC,P_ to V_T_ in OI type III was reduced in seated position, being negative (i.e., paradoxical inward motion during inspiration) and lower compared to OI type IV and controls in supine. In OI type IV patients V_RC,P_ contribution was lower than controls only in seated position. Similarly, the V_RC,A_ contribution to V_T_ was reduced in OI type III patients compared to both OI type IV and controls in both postures. Hence, V_AB_ contribution to V_T_ was higher in patients with OI type III than the other two groups both in supine seated position.

### Chest wall asynchrony

**Figure 5:**
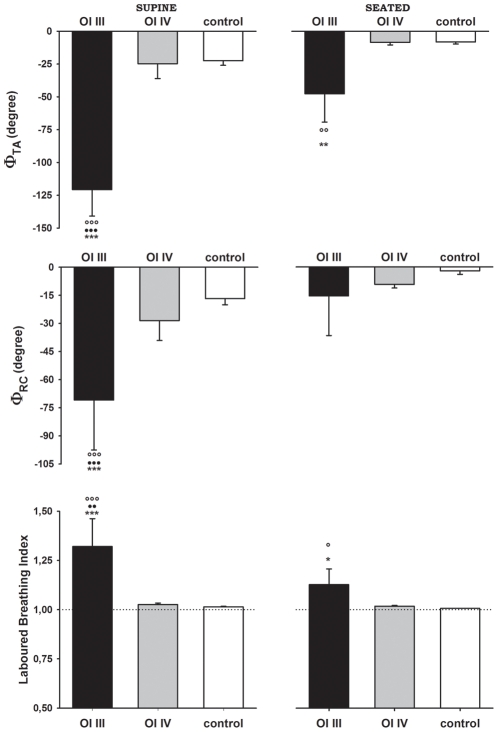
Thoraco-abdominal asynchronies. Average values ± SE of phase angle Φ_TA_ between pulmonary rib cage and abdomen (top panels), phase angle Φ_RC_ between pulmonary rib cage and abdominal rib cage (middle panels) and labored breathing index (bottom panels) in OI type III patients (black bars), OI type IV (grey bars) and control group (white bars) in supine (left panels) and seated (right panels) position. °, °°, °°°: p<0.05, p<0.01, p<0.001 (vs control); *, **, ***: p<0.05, p<0.01, p<0.001 (vs OI type IV); ••,•••: p<0.01, p<0.001 (vs seated).

Patients with OI type III were characterized by high levels of dynamic thoraco-abdominal asynchronies as shown in figure 5. Φ_TA_ was lower in OI type III patients than OI type IV and controls, both in supine and seated position. Φ_RC_ was lower in OI type III patients than OI type IV and controls, but only in supine position. Accordingly, the LBI was higher in OI type III patients than OI type IV and controls, in both positions. In OI type III patients only, Φ_TA_, Φ_RC_ and LBI were significantly different between seated and supine position.

## Discussion

It is known that patients with osteogenesis imperfecta are characterized by bone fragility, frequent fractures, bowing of the long bones, growth disproportionately distributed affecting predominantly trunk height more than the other trunk dimensions, collapsing of thoracic vertebrae resulting in a more horizontal position of the ribs and sternal deformities [1]–[7], [22]. It is not completely clear, however, if and how these features affect the breathing pattern in this disease.

The present study provides for the first time a detailed description of respiratory function in the two non-lethal more severe types of OI. An important original aspect of the present study is that, in addition to standard spirometry, we used the opto-electronic technique to noninvasively assess chest wall geometry, rib cage deformities and thoraco-abdominal kinematics in two different postures.

Several anthropometric parameters are significantly different between OI patients and controls. Only type III patients, however, are characterized by significantly lower angles at the xiphosternal level, both in the sagittal and in the transversal plane. We believe that this altered geometry characterizing pectus carinatum has important consequences in terms of volume variations of the chest wall compartments during breathing. In fact, in six out of seven patients with OI type III, paradoxical inward movement of the pulmonary rib cage was detected during spontaneous breathing at rest in supine position. In seated position, no paradoxical motion was present, however, the percentage contribution of V_RC,P_ to tidal volume was still significantly lower than controls. These results suggest that in OI type III patients the deformed rib cage (i.e., pectus carinatum, more horizontal ribs and more compliant rib cage from brittle bones) alters the normal action of the intercostal muscles and requires the diaphragm to compensate for their reduced contribution to tidal volume. An additional indication that in these patients diaphragm function seems to be preserved is that they do not show significant nocturnal oxygen desaturation.

It can be hypothesized that the reduced angle between the ribs and the sternum modifies the action of the intercostal muscles and alters the “bucket-handle" motion of the ribs in relation to their fulcrum, the sternum [23]. Posture plays an important role not only in the distribution of tidal volume into chest wall compartments but also in the synchrony of their expansion. In our OI type III patients, not only the percentage contribution of V_RC,P_ to tidal volume (Figure 4), but also thoraco-abdominal (Φ_TA_) and rib cage (Φ_RC_) asynchronies (Figure 5) were significantly different between seated and supine position. The generally lower activity of the neck and rib cage muscles (scalene, sternocleidomastoid, and parasternal intercostal) in the supine position [24], which in these patients is combined with the altered geometry of the rib cage, is likely the main reason of these differences.

The rib cage asynchronies and the paradoxical inward motion of the pulmonary rib cage observed in association with the expansion of the abdominal rib cage in OI type III in the supine position also suggest that in these patients rib cage distortions are significant and therefore a substantial fraction of the force developed by the diaphragm on the rib cage would go into distorting it, and only a small fraction into changing chest wall and lung volume. This is an inefficient way to breathe [25] because rib cage distortions, minimal during breathing in normal subjects even during heavy exercise [26], [27], are costly.

An implication of these findings is that rib cage deformities, rather than scoliosis which was present in both OI types, is the determinant of the altered breathing pattern in type III. The correlation between spirometric abnormalities and severe scoliosis in OI has been previously reported [11], [12]. Widmann et al [12] did not find any correlation between kyphosis, chest wall deformities, assessed by radiographic measurements, and FVC. These findings are apparently in contrast with those of the present study, but it should be considered that we analyzed severe and moderate forms of OI as two separate groups and a new method was introduced to dynamically assess chest wall geometry and thoraco-abdominal volume variations without using invasive radiations.

Another original aspect of the present study is the evaluation of the ventilatory pattern. In general, patients with severe osteogenesis imperfecta breathe with rapid and shallow breathing in both positions. The reduced ability of the rib cage compartment to expand is probably the cause of the reduced tidal volume and the concomitant increased breathing frequency could represent the compensatory mechanism to try to guarantee normal minute ventilation. However, this mechanism is not fully effective in the seated position, as shown by significantly lower minute ventilation in OI patients.

The relatively low number of patients could represent a limitation of this work, even if these patients behave very homogeneously. Despite these limitations, we believe that the findings of the present study might have important implications for the functional evaluation and the treatment of OI patients in clinics.

As recently pointed out by Stanojevic et al [28], special attention should be paid on choosing the algorithm for the predicted spirometric values. This is particularly true in Osteogenesis Imperfecta characterized by disproportionally distributed growth. We found that in our patients the standard reference equations proposed by Quanjer et al [13] and largely used in clinics, provide expected values of FVC and FEV_1_ higher than normal. Similarly, Takken et al [22] showed that the reduced patients' height determines higher FVC and FEV_1_ values even in children with OI type I, the mildest form of the disease. These high values of FVC and FEV_1_ are probably artifactual, at least in OI type III, because it is known that cardio-pulmonary insufficiency and respiratory infections represent the leading cause of death in OI. In contrast, the equations proposed by Kuster et al [14] provides values of FVC and FEV_1_, expressed as percentage of predicted values, significantly lower than controls.

In conclusion, in patients with severe OI a direct relationship exists between the structural modifications of the rib cage and the pattern of volume variations during breathing. Patients with OI type III are characterized by pectus carinatum and inspiratory paradoxical inward motion of the pulmonary rib cage during quiet spontaneous breathing in supine position, associated to a high level of asynchrony between the three chest wall compartments. On the other hand, pectus carinatum, paradoxical motion and significant asynchronies between chest wall compartments are not features of type IV patients. These findings suggest that functional assessment and treatment of OI should be differentiated in these two forms of the disease.
